# Anæsthetics

**Published:** 1898-10

**Authors:** H. H. Burchard

**Affiliations:** Philadelphia


### International Dental Journal.

VOL. XIX. OCTOBER, 1898. No. 10.

### Original Communications.[1]

### ANÆSTHETICS.[2]

BY H. H. BURCHARD, M.D., D.D.S., PHILADELPHIA.

FOR purposes of convenient classification, and as an aid to memorizing, I have classed nearly all of the drugs used in dentistry under three heads,—antiseptics, anæsthetics, and astringents. We have discussed the antiseptics, their composition, and their specific effects upon the protoplasm of bacteria and their waste products, together with such chemical features and properties of each substance as give them distinctiveness.

To-day we begin the study of the second class,—the second group,—classified under the head of anæsthetics. You will find in your note-books that I have defined anæsthetics as agents which diminish the reception, transmission, or perception of impressions which cause pain.

There is another word applied to agents which have the power of preventing or abolishing pain,—*i.e.*, analgesics. These words are frequently used interchangeably, but they are not entirely synonymous.

---

[1] The editor and publishers are not responsible for the views of authors of papers published in this department, nor for any claim to novelty, or otherwise, that may be made by them. No papers will be received for this department that have appeared in any other journal published in the country.

[2] Report of two of a series of lectures on pharmacology.

The word anæsthesia was coined by the late Oliver Wendell Holmes, for many years the professor of Anatomy in the Harvard Medical School, to designate the state of insensibility produced by the inhalation of ether. You will see, therefore, that it implies a condition associated with loss of consciousness. Its etymology is from the Greek words *an*, without, and *aisthesis*, sensation; so that the word is admirably descriptive. An analgesic, strictly defined, is an agent which removes existing pain, *an*, without, and *algos*, pain.

There is no implied or stated loss of consciousness in the content of this word. It would seem, then, to be preferable to apply the term anæsthetics to those agents whose administration prevents pain by abolishing consciousness; analgesics, to those agents which do not cause a loss of consciousness. We will take up first the study of the agents which we have called general anæsthetics.

At the very beginning of this study we are confronted with two words about which there is much diversity of opinion as to definition. These are pain and consciousness; now the definition of these words depends largely upon the point of view,—that is, the way we look at them.

We will ignore entirely the metaphysical side of the question, which can lead to nothing but confusion and an ultimate confession of ignorance,—that is, relative ignorance. Metaphysical research has, as a matter of fact, but little if any practical value in the study of the sciences, excepting perhaps that of theology. Its study has, however, this virtue, it teaches us the limitations of human knowledge, but utterly fails to give us nineteenth and twentieth century science. In the same vein, we will not view the subject of consciousness as the metaphysician would view it, but say at the outset that for our present purposes pain and consciousness are things to be studied in the physiological laboratory. First for a definition of pain: we cannot define it absolutely, so let us call it a degree or condition of sensation which causes physical discomfort. Here again is a difficulty, we have the word sensation which requires explanation and study. It implies that the individual is conscious,—*i.e.*, has consciousness of something very wrong with the body at some point or other. You see how we are driven back to a study of consciousness. When we speak of consciousness in man, we mean his recognition of his relations with external objects through the agency of the five senses. It might be objected to here, that a man might have lost all five of his senses and yet be conscious. To this objection may be retorted, How can we possibly tell that consciousness exists in such a person, and, secondly, that such a man has not consciousness as we understand it, for his state of mind is governed entirely by old, not new, impressions made upon his brain-cells? We are accustomed to associate all thinking, all consciousness, with the presence of a brain,—that is, a cerebrum; but any physiologist could point out the fallacy of this; for while, of course, we cannot positively affirm it, it is probable that even mass of living matter, be it an amœba, a radiate, a mollusk, a fish, or a mammal, has in some degree the quality which, in its most highly evolved condition, is the consciousness of man.

The physiologist recognizes that before we can have sensation or a knowledge of surroundings three things are essential. First, a nerve-centre or centres in which consciousness or sensation is actually formed; secondly, a means of receiving external impressions; and, thirdly, a line of connection between these two; that is, there must be a perceptive centre, a receptive apparatus, and a transmitting apparatus. In studying the subject of anæsthetics in relation to man, this means that in sensation there are involved a perceptive apparatus, situated in the outer portion of the cortex of the brain's greatest ganglia, the cerebrum; next, a receiving apparatus, or the endings of nerves of sensation and nerves of special sensation; next, the nerve-paths connecting these two, the centre and the periphery. Sensation of any kind consists in the reception by the endings of special (sensory and special sense) nerves, the transmission of this sensation along nerves and up the spinal cord, thence to the cortex of the brain. So long as the terminals of the nerves, the sensory nerve-endings, receive only stimuli or impulses measured within an unknown amount, the impulses are carried to the brain, and produce what is called normal sensation. That is, let us say, a sharpened point is placed against the finger and pressed upon by a gradually increasing weight, until an ounce in weight is used; this produces no actual discomfort, the nerve terminals are not over-stimulated, are not irritated. Increase the weight to, say, four ounces, and a feeling of discomfort is noted, the terminals are over-stimulated, and the impressions carried to the brain are translated into pain.

The existence of the pain, we say then, is determined by the function of brain-cells.

It has been shown by Ferrier, in experiments upon monkeys, that if we cut out the posterior portion of the cerebrum (near the hippocampus), the monkey is incapable of the touch sense; and when its surface, the ends of its sensory nerves, is irritated, the animal gives no evidence of pain, showing that the pain sense is located in this portion of the brain. It is in the cortex of the brain, also, that physiologists and psychologists believe the numerous centres which preside over idea-formation, imagination, and all of the higher faculties are located. Now, of course, if we remove or destroy these areas, function will be destroyed. So that if we remove the portion of the brain called the tactile centre, we prevent sensation and pain,—that is, we can prevent pain by destroying the perceptive centre.

Again, if we destroy the ends of the sensory nerves, they cannot receive impressions, hence we can prevent pain by destroying the receptive apparatus.

Still more, if we have both perceptive centre and receptive nerve-endings healthy, and cut or destroy the pathway between perceptive centres and sensory nerve-endings, although the ends of these nerves may be irritated, there can be no pain, because the irritation cannot be carried to the pain-centre,—that is, we may prevent pain by destroying transmission.

To recapitulate: we may induce a condition of anæsthesia, may prevent pain, first, by preventing the reception of painful impressions; secondly, by preventing their transmission; thirdly, by preventing their perception.

If we destroy the function of the perceptive centre, we have a general abolition of sensation, a general anæsthesia. If we destroy the function of the transmitting apparatus,—the spinal cord,—it will depend upon the point of the injury what the extent of the anæsthesia will be; for example, if the spinal cord were cut above the lumbar region, we would have paralysis of the lower extremities; if cut in the cervical region, of the upper extremities also. The receptive apparatus, when destroyed, is only destroyed over a comparatively small surface, hence this would be followed by anæsthesia (local) of that region alone. Now it is not necessary that these parts, any of them, be actually destroyed to prevent them performing their function. For example, the perceptive centre is made up of special nerve-cells, which, when in normal condition, preside over or perform the function named. To perform their function they must have what any and all cells must have, the proper conditions of life,—that is, they must receive a proper food-supply, including oxygen, their waste material (the ashes of the vital furnace) must be removed, and of course they must be healthy in themselves. If we interfere with their vitality and their nutrition, we interfere with their function. If we deprive them of oxygen or food, we destroy their function; again, if we establish such a condition in them that they cannot take up oxygen, we destroy their function. Can this be done? Yes; and the agents which are capable of doing it are called general anæsthetics.

Having discussed the conditions existing as a basis, and the object to be obtained, the next step is a study of the agents which can accomplish this object. The principal ones of this group of general anæsthetics are chloroform, ether, and nitrous oxide.

To have an intelligent idea of how these agents act, it is essential that we should examine their composition. Let us take up, first, chloroform. You will notice by its chemical formula, $CHCl_3$, that it belongs to what your professor of chemistry tells you is the monocarbon series.

This series begins with the body called marsh gas, or methane, its formula being $CH_4$. This gas, when inhaled, is found to lessen the function of the perceptive centre. Replace one of the hydrogen atoms by chlorine, and we have methyl chloride, which has a like property, but more pronounced; instead of having now a heavy gas we have a very light and volatile fluid: we shall speak again of this substance. Substitute two chlorine atoms, and we have bichloride of methylene, $CH_2Cl_2$, a stronger anæsthetic and a heavier fluid. Replace three hydrogen atoms by three of chlorine, and there is formed a powerful anæsthetic and a still heavier fluid, $CHCl_3$, or chloroform. Displace all of the hydrogen by chlorine, and we have $CCl_4$, tetrachloride of carbon, a heavier fluid and an anæsthetic. Methane $CH_4 - H = CH_3$, or methyl; methyl $+ Cl = CH_3Cl$, methyl chloride. $CH_4 - H_2 = CH_2$, or methylene; methylene $+ Cl_2 = CH_2Cl_2$, methylene bichloride. $CH_4 - H_3 = C$, or formyl; formyl $+ Cl_3 = CHCl_2$, formyl trichloride or chloroform. $CH_4 - H_4 = Cl + Cl_4 = CCl_4$, tetrachloride of carbon. While, as stated, all of these substances have anæsthetic power, these are by no means all equally useful in this field. Let us examine the vapor density: The greater the weight of the molecule of any of these substances the less volatile it is, and the more prolonged will be its effects upon the nervous system. Beginning with $CH_4$, we have a molecular weight of 16; with chloride of methyl, a molecular weight of 47.5; with chloroform the weight is 118.5. Each atom of chlorine (35.5) as it replaces hydrogen (H) adds greatly to the density of the substance and increases its vapor density and boiling-point. So that when inhaled the effects of these agents will be prolonged according to the number of chlorine atoms they contain. With the base of methane, $CH_4$, we can construct still other anæsthetics; instead of replacing hydrogen atoms by chlorine, bromine may be the element substituted. Thus, $CH_4 - H = CH_3$ or methyl; $CH_3 + Br = CH_3Br$, methyl bromide.

When $CH_4 - H_3$, or formyl, is combined with $Br_3$, we have $CHBr_3$, formyl bromide, called bromoform; both of these substances are anæsthetics. The text-books state that the effects of bromoform are much more transient than those of chloroform, but if we examine its molecular weight, $CHBr_3 - 12 + 1 + 239.25 = 252.25$, and compare it with that of chloroform, $CHCl_3 - 12 + 1 + 106.5 = 119.5$, we see that there has probably been some error of observation as to its effects. We may also form anæsthetics by substituting iodine for hydrogen in the methane nucleus, $CH_4 - H =$ methyl; $CH_3 + I = CH_3I$, methyl iodide. If we replace three hydrogen atoms by three of iodine, we have $CH + I_3 = CHI_3$, or formyl triiodide, iodoform. We see that here we have, instead of a volatile fluid and a body of low molecular weight, a very heavy molecule and a solid body,—$CHI_3$ ($12 + 1 + 381 = 394$).

The next member of our anæsthetic agents is ether or ethyl oxide; it is derived from the dicarbon series,—$C_2H_6$ = ethane. Ethane deprived of an atom of hydrogen becomes $C_2H_5$, ethyl; this behaves as does methyl as a monovalent radical, entering into combination with chlorine, bromine, and iodine, and forming a second class of anæsthetics, $C_2H_5 + Cl$ = ethyl chloride ($24 + 5 + 35.5 = 64.5$), a very volatile fluid, and, although a general anæsthetic, you will see from its molecular weight that it is too volatile for practical use: we will refer to it again under the head of local anæsthetics. $C_2H_6 - H_2$ = ethylene, $C_2H_4 + Cl_2 = CH_4Cl_2$, or ethylene bichloride, called Dutch liquid, an anæsthetic having theoretically great usefulness, but it has been used but little. The combination with iodine, $C_2H_5I$, although a strong anæsthetic, is not used for this purpose. The combination with bromine, $C_2H_5Br$ (ethyl bromide), is a powerful anæsthetic, which has had limited use.

The interesting and important member of this group is ethylic ether, $(C_2H_5)_2O$, ethyl oxide. It may aid you in remembering the chemical composition of this substance by regarding it as a substitution product of water. Let us represent water graphically $H - O - H$, two atoms of monovalent hydrogen held in combination by the two bonds of bivalent oxygen. Replace one monovalent element by the monovalent radical $C_2H_5$, or ethyl, and we have $(C_2H_5) - O - H$, ethyl hydroxide or alcohol. Replace both hydrogen atoms by two molecules of ethyl, and we have $(C_2H_5) - O - (C_2H_5)$ = ethyl oxide or ethylic ether.

The last member of our series is nitrous oxide, or nitrogen monoxide. If you will observe a person who is being anæsthetized by nitrous oxide you will be struck by the livid appearance of the face, the bluishness of the lips, and ghastly look about the eyes; from your studies in physiology you could recognize the condition to be what is called cyanotic; that there appears to be an absence of oxygen or an excess of carbon dioxide in the blood, and one might jump to the conclusion that this was the cause of the anæsthesia, as indeed it might be; but if you will admit, after the Thomas method, atmospheric air with the nitrous oxide, still anæsthesia will be produced, although more slowly. Admit oxygen, after the Hewitt method, and while there is an entire absence of the cyanotic appearance, anæsthesia is brought about. We are then driven to the conclusion that nitrous oxide has a specific anæsthetic effect, operating upon cerebral nerve-centres. We pass next to a consideration of how these agents act upon consciousness and sensation. First and foremost, what are their probable cellular effects; next, what are their gross and visible effects.

There is no better or more rational explanation at present than that known as the semi-coagulation theory of Claude Bernard. That is, it has been shown that certain agents, such, for example, as morphine (Binz), have the power of causing a contraction of nerve-cells, similar to that caused by quinine upon white blood-corpuscles; a form of spasm of the cell occurs, a fixation of the protoplasm which is probably the first stage of coagulation, and it is probable that alcohols, chloroform, and ethers have a similar power,—that is, they interfere with the normal function of brain-cells. Applied in small doses, they stimulate, next over-stimulate, and next paralyze, for the time being, the function of these cells. The probable mode of action is that of a combination of the anæsthetic, carried to the cells, in loose chemical bonds with the protoplasm, or at least a portion of the cell contents. While this union persists, the cell cannot oxidize properly,—*i.e.*, cannot functionate,—and waste products of cell action are not removed. As soon as the weak chemical bonds are dissociated the function of the cell returns. The length of time the effects persist depends upon the volatility, the vapor density; hence the molecular weight of the agent employed; the lower the molecular weight the more rapidly the substance is eliminated, the more transient is the anæsthesia; and *vice versa*, the greater the molecular weight the more prolonged the anæsthesia; thus a light gas ($N_2O$) is much more transient in its effects than the heavy vapor of chloroform. An anæsthetic taken in liquid form has still more prolonged action; the anæsthetic solids are usually impracticable as true anæsthetics, but are used in small doses as anodynes.

If you will watch a person carefully who is being anæsthetized, you will note that the highest mental functions are the first to be affected. Imagination and kindred functions are the first to succumb; next, the special senses are affected in the order of their nerve emergence; next, lower functions succumb, until all consciousness is gone, and the respiration and circulation continue, although affected. Increase the amount of the drug and soon there is evidence that the nervous centre of respiration is paralyzed, respiration ceases; continue the administration and the heart-centre is paralyzed, and death results.

When respiration ceases, of course oxidation ceases, and if the asphyxia is prolonged beyond very few minutes death results. It has been taught and shown you how the muscular movements of respiration may be simulated,—that is, how to practise artificial respiration. This is a subject of much importance, for even though the centre of respiration be paralyzed, if we can cause the expansion and contraction of the chest cavity, the ingress of supplies of oxygen will continue; and as this supply is intimately concerned with ridding the nerve-centre of the anæsthetic, the importance of artificial respiration becomes evident. If we can maintain this artificial respiration until the cells free themselves of the drug, the respiratory centre resumes its function, as do the other centres in the reverse order of their affection. The more volatile the agent employed the more quickly it is eliminated, hence ether is gotten rid of more quickly than chloroform.

Leaving now the discussion of agents which produce anæsthesia by abolishing perception, we pass to the second group, those which prevent the reception of impulses which would cause pain.

There are drugs which, when administered, lessen the function of the terminals of sensory nerves, without abolishing consciousness or inducing sleep; these are various narcotics; their discussion is reserved for another time. The agents which belong to our present study are those known as local anæsthetics. Of course, the reception of impressions may be prevented by destroying the receptive apparatus, by cutting or burning, for example; heat or cauterizing chemicals will bring about this end; but this is not inducing local anæsthesia. Just here is another distinction between the words anæsthesia and analgesia; an anæsthetic implies a temporary cessation of sensation, but in analgesia we imply that pain is prevented by any means available; thus carbolic acid applied to the skin to render painless the opening of an abscess is an analgesic, but it destroys the tissue to which it is applied; zinc chloride applied to dentine has an analgesic effect, but it destroys instead of holding in abeyance the function of the receptive apparatus. The function of the receptive apparatus may be lessened by applications of cold, as by applying a mixture of ice and salt to any part; one of the most noticeable effects of accidental freezing of the extremities, as the toes or ears, is an entire absence of sensation in the frozen part. You must remember, however, that after freezing, an inflammatory reaction, often violent, occurs when the temperature of the parts is raised, and ulceration commonly results (chilblain) in consequence. It is to prevent this violent vascular reaction that frozen parts are rubbed with snow instead of being warmed.

The volatile liquids, methyl chloride, $CH_3Cl$, and ethyl chloride, $C_2H_5Cl$, directed in a spray against any part, will by their rapid evaporation produce the same effects as freezing. Any highly volatile substance will have the same effect; a spray of alcohol, $CH_5HO$, of ether $(C_2H_5)_2O$, of chloroform, $CHCl_3$, or of liquid nitrous oxide, $N_2O$, will cause freezing, the rapidity and extent of the freezing being governed by the volatility of the agent applied.

We find among drugs many agents which are capable of lessening the irritability and reducing the function of the terminals of the sensory nerves, as, for example, the alkaloids veratria and aconitia, which are often applied in ointment to relieve painful affections of the fifth cranial nerve. You will find use for them in dentistry to relieve the pain, attending difficult eruption of lower third molars; we will discuss their mode of action at some other time. Many of these agents exhibit before their anæsthetic effect an irritant action, which has led Liebreich to call the effect produced by them "anæsthetica dolorosa." The interesting members of this group are the benzoyl derivatives, the principal of which is cocaine. We find among several of the local anæsthetics an interesting chemical relationship full of significance, there being a group of substances the members of which possess the power of lessening the function of sensory nerve terminals, which form a chemical chain. First, atropine, the tropate of tropine: this drug possesses the power of paralyzing certain nerve terminals; combine with tropine, benzoic acid instead of tropic acid, and we have benzoyl tropine, or homatropine, which has a marked local anæsthetic action.

The next substance also contains the benzoyl and tropic elements: it is benzoyl pseudotropine, or tropacocaine; all three of these agents are irritant anæsthetics, causing some degree of hyperæmia when applied to a mucous surface. To the benzoyl nucleus add methyl and ecgonine, and we have methyl benzoyl ecgonine, or cocaine.

Cocaine is, by boiling, split up into methylic alcohol, benzoic acid, and ecgonine. The leaves of coca contain another substance in which isatropyl replaces benzoyl, forming a body known as isatropylcocaine, a cardiac poison. Another agent has been recently introduced among local anæsthetics, containing benzoyl and methyl, it is called eucaine. It was its chemical composition which indicated that it would act as a local anæsthetic. Unlike cocaine, this substance is not split up, decomposed, by boiling in distilled water. The chain as we now have it is,—

| | |
|---|---|
| Atropine . . . . . . . . . | the tropate of tropine. |
| Homatropine . . . . . . . | benzoyl tropine. |
| Tropacocaine . . . . . . . | benzoyl pseudotropine. |
| Cocaine . . . . . . . . . . | benzoyl methyl ecgonine. |
| Eucaine . . . . . . . . . . | benzoyl methyl-tetramethyl ester +. |

Any of these agents, placed on a mucous surface, will, after a period, paralyze the sensory nerve terminations,—that is, will produce local anæsthesia. The last three members are most marked in power. Tropacocaine and eucaine both produce more or less dilatation of the vessels (hyperæmia) of the parts. Cocaine causes contraction of the vessels (ischæmia) of a part. This has an important clinical bearing. It is when these drugs are taken internally, by the the stomach or by hypodermic injection, that we find the greatest importance in regard to their effects. For example, while in large doses tropacocaine, cocaine, and eucaine all cause paralysis of the respiratory centre, the paralyzant action, hence danger, is much more marked with cocaine than with either of the other two. When either of these substances is injected into a part by means of a hypodermic syringe, a comparatively wide area of insensibility is found. When injected about the trunk of a nerve, a portion of the transmission pathway, insensitivity and anæsthesia will be found in the area supplied by the terminals of the nerve; for example, injected deep down upon the inner and posterior portion of the inferior maxillary bone, a wide area of anæsthesia results,—that is, it paralyzes the power of transmission, so that cocaine can cause anæsthesia by abolishing reception and transmission; more than this, if applied to the cortex of the brain, it can paralyze the nerve-centres and prevent perception.

The succeeding group of agents includes those which induce anæsthesia by preventing the transmission of painful impulses from the receptive to the perceptive apparatus. We have seen that cocaine possesses this power, as do also many of the general anæsthetics, if applied to nerve-trunks. Dr. Roberts Bartholow several years ago introduced the practice of injecting chloroform deep about the trunk of the sciatic nerve for the relief of sciatica.

A group of agents having the power of suspending transmission include substances resembling alkaloids which have been introduced in the past fifteen years; they are known as the coal-tar derivatives. These are extremely interesting bodies; they resemble alkaloids in effects and chemical composition; they may be regarded as substitution compounds of ammonia,—that is, ammonia ($NH_3$) written graphically,

$$N \begin{cases} H \\ H \\ H, \end{cases}$$

may have one, two, or three hydrogen atoms replaced by one or more organic radicals:

$$N \begin{cases} H \\ H \\ C_6H_5. \end{cases}$$

Replacing one hydrogen atom by benzene ($C_6H_5$), we have aniline. Replacing one hydrogen atom by phenyl ($C_6H_5$), another by acetyl $CO.CH_3$ or ($C_2H_3O$), we have

$$N \begin{cases} H \\ C_6H_5 \\ CO.CH_3, \end{cases}$$

forming phenylacetamide, or acetanilide, commonly called antifebrine. If we replace the remaining hydrogen atom by $CH_3$, or methyl, we have

$$N \begin{cases} CH_3 \\ C_6H_5 \\ CO.CH_3, \end{cases}$$

another substance called methyl acetanilide or exalgin. The introduction of the methyl radical increases the analgesic power. These may be taken as types of these compound ammonias, all of which have the power of reducing the transmission of painful impressions. This introduces the principle of foretelling the probable effects of drugs which may be made in the laboratory, built up, synthetic drugs, by knowing their chemical composition. You will hear much of this phase of pharmacology from now on.

